# Near-infrared roll-off-free electroluminescence from highly stable diketopyrrolopyrrole light emitting diodes

**DOI:** 10.1038/srep34096

**Published:** 2016-09-28

**Authors:** Mauro Sassi, Nunzio Buccheri, Myles Rooney, Chiara Botta, Francesco Bruni, Umberto Giovanella, Sergio Brovelli, Luca Beverina

**Affiliations:** 1 Dipartimento di Scienza dei Materiali Università degli Studi di Milano-Bicocca, via Cozzi 55, 20125, Milano, Italy; 2 Istituto per lo Studio delle Macromolecole, Consiglio Nazionale delle Ricerche (ISMAC-CNR), Via Bassini 15, 20133, Milano, Italy

## Abstract

Organic light emitting diodes (OLEDs) operating in the near-infrared spectral region are gaining growing relevance for emerging photonic technologies, such as lab-on-chip platforms for medical diagnostics, flexible self-medicated pads for photodynamic therapy, night vision and plastic-based telecommunications. The achievement of efficient near-infrared electroluminescence from solution-processed OLEDs is, however, an open challenge due to the low photoluminescence efficiency of most narrow-energy-gap organic emitters. Diketopyrrolopyrrole-boron complexes are promising candidates to overcome this limitation as they feature extremely high photoluminescence quantum yield in the near-infrared region and high chemical stability. Here, by incorporating suitably functionalized diketopyrrolopyrrole derivatives emitting at ~760 nm in an active matrix of poly(9,9-dioctylfluorene-alt-benzothiadiazole) and without using complex light out-coupling or encapsulation strategies, we obtain all-solution-processed NIR-OLEDs with external quantum efficiency as high as 0.5%. Importantly, our test-bed devices show no efficiency roll-off even for high current densities and high operational stability, retaining over 50% of the initial radiant emittance for over 50 hours of continuous operation at 10 mA/cm^2^, which emphasizes the great applicative potential of the proposed strategy.

Organic light emitting diodes (OLEDs) are among the most successful examples of organic-based devices actually meeting commercialization criteria^1^,^2^,^3^,^4^,^5^. Efficient devices are available for all primary colors, thus making organic displays a market reality. Broadband white light emission, which is crucial for interior lighting applications, has also been demonstrated with a variety of organic materials and device architectures^6^,^7^,^8^. Full compatibility with large-area flexible substrates is also a consolidated result^9^.

Recent years witnessed growing interest in the development of efficient OLEDs operating in the near-infrared spectral region (NIR, 700–2500 nm), which is a portion of the electromagnetic spectrum extremely relevant for applications spanning from bioimaging, photodynamic therapy and night vision to telecommunications and sensors^10^. The achievement of efficient light emission in the NIR from organic lumophores is, however, a relevant problem in itself, since not only electroluminescence (EL) but also standard photoluminescence (PL) becomes progressively inefficient upon narrowing the energy gap from the visible to the IR region. Lanthanide chelates (Er, Nd, Pr, Tm, Yb most notably) are considered amongst the most efficient organic-based NIR emitters^11^,^12^,^13^,^14^,^15^,^16^,^17^,^18^, however, the EL quantum efficiency (EQE) of NIR-OLEDs based on these compounds is still low^19^,^20^,^21^. Recently, two other material systems have emerged as possible candidates for NIR-OLEDs: phosphorescent metal complexes (i.e. Ir, Cu, Pt) and donor acceptor (D-A) compounds. Within the first group, the use of a Pt-benzoporphyrin provided a remarkable EQE of 9.2% at 773 nm^22^. On the other hand, copper-phtalocyanines embedded in a 4,4′-N,N′-dicarbazole-biphenyl matrix enabled EL at 1100 nm although with lower efficiency^23^. Solution- and vacuum-manufactured devices emitting around 700–800 nm based on Ir(III) metal complexes have reached efficiencies up to ~3%^24^,^25^. Recently, Wang *et al*. exploited the thermally activated delayed fluorescence of a phenanthrene derivative to fabricate OLEDs with 2.1% efficiency at 710 nm^26^.

To date, the only metal-free organic systems enabling NIR-EL are D-A compounds^27^,^28^,^29^,^30^. In these systems, the combination of strong electron-donating and electron-withdrawing substituents at the opposite ends of easily polarizable conjugated moieties is a well established concept providing organic molecules with energy gap well within the NIR region. It should be noted, however, that strong HOMO-LUMO charge transfer transitions usually result into low emission efficiency^31^. The literature reports only a few examples of completely organic structures providing sizeable NIR-EL. The best result to date pertains to “butterfly shaped” D-π-A-π-D molecules featuring broad emission spectra (600–900 nm) peaked at 700 nm which enabled to obtain EQE > 1.5%^29^. Electroluminescence above 1000 nm from D-A molecules was also demonstrated with EQE = 0.76%^32^.

In this work, we demonstrate, for the first time, efficient and ultra-stable NIR-OLEDs based on diketopyrrolopyrrole derivatives. These organic systems, of general formula **2** (Supplementary Scheme 1)^33^,^34^,^35^, feature a simpler and more compact chemical structure with respect to D-A compounds, extremely strong optical absorption, facile and scalable synthesis and rank amongst the most efficient emitters in the 700–800 nm spectral region. Furthermore, they share with the corresponding diketopyrrolopyrrole precursors the remarkable chemical stability typical of high performing pigments.

In order to enable solution-processing and to reduce aggregation-induced PL quenching in close packed films^28^, we included in the **DPPcy** structure two dimethyloctyl solubilizing chains (Fig. 1a). The **DPPcy** derivative was prepared starting from the DPP precursor **DPP-CN** according to a slightly modified procedure with respect to the general protocol described by Daltrozzo *et al*. (Supporting Information)^33^,^34^,^35^. **DPPcy** is a crystalline solid, freely soluble in chlorinated solvents (solubility in chloroform above 20 mg/ml), which makes it mixable with conjugated organic polymers and easily processable through common wet deposition methods. Figure 1b shows the optical absorption and the continuous wave (cw) PL spectra of **DPPcy** spin-coated on a glass substrate. As expected from cyanine-like compounds, **DPPcy** features a narrow, vibrationally resolved, absorption band with maximum at ~760 nm. The compound is characterized by efficient NIR emission peaked at ~780 nm, with a small Stokes-Shift from the respective absorption onset and a remarkable quantum yield of ~48 ± 6% in the solid state.

A schematic of the device structure and corresponding flat-band energy diagram with respect to the vacuum level are shown in Fig. 1c,d. The device consists of a patterned indium tin oxide (ITO) coated glass substrate with a 40 nm poly(3,4-ethylenedioxythiophene) polystyrene sulfonate (PEDOT:PSS) hole-injecting layer and a 50 nm poly(vinylcarbazole) hole transport layer that further serves to constrain, inside the active layer, electrons injected in the device from an Al-capped Ba cathode (4 nm Ba; 80 nm Al). The emitting layer is a 80–90 nm thick film of a binary blend of **DPPcy** and poly(9,9-dioctylfluorene-*alt*-benzothiadiazole) (F8BT), as discussed below. The energy of the HOMO and LUMO levels of **DPPcy** was estimated using cyclic voltammetry (CV) and differential pulse voltammetry (DPV) in a 2:1 CH_3_CN:CH_2_Cl_2_ solution with 0.1 M TBAClO_4_ as the supporting electrolyte.

Figure 2a shows a comparison between the CV traces of **DPPcy** and its corresponding ligand **DPP-CN**. The redox behaviour of **DPP-CN** is essentially irreversible and very rich. At reductive potentials, two weak and irreversible peaks (at DPV peak potential of −0.43 and 0.74 V vs Fc/Fc^+^ respectively) can be connected with the irreversible reduction of the pyridium salt tautomeric form of **DPP-CN**. This assignment is supported by the complete disappearance of such peaks in the **DPPcy** DPV trace. At more reductive potentials, two additional peaks (at −1.34 and −1.78 V vs Fc/Fc^+^) correspond to the successive reductions of the two DPP cyano-vinyl units. At oxidative potentials, one semireversible and one irreversible peak can be distinguished. The first peak (resolved in the DPV trace in two peaks at + 0.21 and + 0.31 V vs Fc/Fc^+^ respectively, possibly due to conformational equilibrium, again disappearing upon complexation with BF_3_) is associated with the oxidation of the first donating phenoxy group, followed at + 0.56 V by the second one. Unlike **DPP-CN**, the boron complex **DPPcy** possesses a reversible behaviour both in oxidation and in reduction. In details, at reductive potentials two reversible peaks (at −1.11 V and −1.71 V vs Fc/Fc^+^) are associated with the reductions of the DPP cyano-vinyl electron poor moieties. At oxidative potentials one reversible peak at 0.43 V vs Fc/Fc ^+^ can be associated with the oxidation of the first phenoxy group. The oxidation of the second phenoxy unit is barely detectable at around 0.71 V. The direct comparison between the DPV traces indicates that the introduction of the highly electronegative -BF_2_ residue rigidly shifts both the HOMO and the LUMO levels by 0.22 eV, leaving the electrochemical gap (as well as the optical gap) unchanged. Derivative **DPPcy** proved to be suitable for OLEDs also in terms of its thermal properties, as it is highlighted by the TGA traces in air and under nitrogen in Supporting Fig. S1.

For the fabrication of efficient NIR-OLEDs, fluorescent or phosphorescent emitters are typically dispersed or co-evaporated in a conjugated matrix, so as to increase the film homogeneity and to prevent quenching by intermolecular aggregation. Poly(vinylcarbazole) (PVK), with hole transporting ability, has been successfully employed, in combination with electron transporting additives, as a host for NIR-emitting Ir(III) complexes^25^. Upon charge injection, excitons are either generated in the host matrix and successively transferred via Foerster energy transfer (FRET) to the NIR emitter, or they can be generated directly in the emitter after charge localization. In our case, the HOMO and LUMO energies of F8BT (Fig. 1c) are better positioned with respect to the frontier levels of **DPPcy** than PVK. Accordingly, devices fabricated with the same architecture but employing PVK as active matrix show systematically poorer performances than LEDs incorporating FTBT:**DPPcy** blends (Supporting Figs S2 and S3). One further advantage of F8BT is that its hole-transport character can be modified by post-deposition processes, such as thermal annealing above the glass transition temperature (130 °C)^36^,^37^. This has been shown to enhance the EQE of OLEDs by enabling bipolar conduction and by improving the charge balance in the active layer. In addition, such post-deposition tunability simplifies the formulation of the active layer mixture and removes the need for additional charge transport additives^36^.

For these reasons, in our NIR-OLEDs, we employed F8BT as active matrix and doped it with **DPPcy**. Different F8BT:**DPPcy** blending ratios were initially tested to optimize both the film quality and the optical properties (full details reported in Fig. S5 of the Supporting Information). In Fig. 3 the optical properties of the blend with the optimized F8BT:**DPPcy** ratio of 85:15 wt% are shown for a 80–90 nm thick film prepared by spin-coating from a 50/50 vol. toluene/chlorobenzene mixture. The blend shows optical features of both F8BT and the **DPPcy**. Thermal treatment at 130 °C for 15 minutes in nitrogen atmosphere led to phase segregation causing a slight blue-shift of the **DPPcy** absorption spectrum with an increase in intensity of the high energy peak at ~660 nm. Concurrently, the emission peak at ~760 nm broadens and weakens with respect to the F8BT emission at 535 nm. The PL quantum yield of the as-spun and annealed blends are 50 ± 5% and 55 ± 7%, respectively. The change in the PL profile upon annealing is ascribed to reduced FRET efficiency from F8BT to **DPPcy** in the annealed film, in agreement with the formation of phase segregated domains. This scenario is supported by time-resolved measurements of F8BT PL showing stronger acceleration of the decay dynamics of pure F8BT (τ_F8BT_ = 845 ps) in the as-spun blend (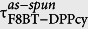 = 65ps, where the subscript indicates that F8BT is in the presence of the **DPPcy** acceptor) with respect to the annealed film (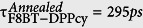)^38^,^39^,^40^,^41^. Within the approximation that FRET is the only additional non-radiative decay channel present in the blends with respect to the pure components, we estimate the FRET efficiency as η_FRET_ = 1 − τ_F8BT − DPPcy_/τ_F8BT_, and find it to be 0.93 for the as-spun blend and 0.66 in the annealed film.

In order to directly monitor the evolution of the surface morphology upon thermal treatment, we performed atomic force microscopy (AFM) and confocal fluorescence measurements. The comparison between height AFM images measured in semi-contact mode of as-spun and annealed 85:15 F8BT:**DPPcy** films (Fig. 3c,d) evidences increased root mean square roughness from 0.34 ± 0.02 nm to 1.46 ± 0.02 nm. Thermal annealing induces phase segregation of F8BT- and **DPPcy**-rich domains, as highlighted by the phase images (inset of Fig. 3c,d) showing dark and bright nanometer sized domains, whose texture is evident in the treated film. Fluorescence microscopy images (Fig. 3e,f) further confirm the formation of segregated domains in the treated film, with brighter F8BT-rich phases, in agreement with the reduced quenching due to FRET to **DPPcy** moieties. Weaker homogeneous green emission is, in contrast, observed in the untreated F8BT film.

To experimentally validate the suitability of **DPPcy** for NIR-OLEDs, we fabricated devices in the ITO/PEDOT:PSS/PVK/F8BT:**DPPcy**/Ba/Al architecture embedding F8BT:**DPPcy** films with different blending ratios by successive spin coating of the organic layers and by thermal evaporation of the metal cathode. A thin layer of hole injecting/electron blocking PVK was spin-coated on PEDOT:PSS in order to confine excitons far from the anode and thereby promote radiative recombination^42^.

The EL spectrum of the **DPPcy**-based LEDs is shown in Fig. 4a, together with a photograph of a working device taken with a UV-Vis filtered IR-camera, showing uniform illumination across the whole electrode (5.4 mm^2^). The EL switches on at 8V and is fully located in the NIR spectral region 700–900 nm with maximum at 753 nm (FWHM of 65 nm) with no contribution by F8BT. We highlight that our devices are manufactured exclusively by solution method. The obtained maximum EQE = 0.55% at 2 mA/cm^2^ and maximum radiant emittance (R_e_) of 200 mW/m^2^, achieved with annealed 85:15 wt% F8BT:**DPPcy** active layers (Fig. 4c,d), not only outperform literature solution-processed NIR OLEDs, but they are also competitive with the performances of devices incorporating sublimated molecular multilayers. The difference between the PL and EL profiles (Figs 3a and 4a) is consistent with the preferential generation of excitons in **DPPcy** molecules by charge trapping from F8BT (see the energy diagram in Fig. 1d) with respect to the distributed exciton generation under optical excitation, which leads to intense F8BT emission at ~550 nm (Fig. 3a). The role of charge trapping as excitation source for **DPPcy** under electrical drive is corroborated by considering that, in the annealed film, η_FRET_ = 0.66 (Fig. 3b), which indicates that FRET alone cannot account for the observed EL spectrum due exclusively to **DPPcy** at driving voltage above the energy gap of F8BT (V = 10 V, Fig. 4a). The annealing process, performed prior to the deposition of the cathode, is crucial for achieving the observed LED performances, as it leads to a 30-fold enhancement in EQE with respect to devices embedding pristine active layers (Fig. 4c). For direct comparison, in the inset of Fig. 4b, we report the current density-voltage-radiant emittance response of an OLED embedding a pristine F8BT:**DPPcy** blend, showing a turn-on voltage as high as 16 V and ten times lower emittance with respect to the annealed device despite over 4-fold higher current density.

NIR-OLEDs with blending ratios ranging from 90:10 wt% to 70:30 wt% show systematically lower efficiency with respect to devices with blending ratio of 85:15 wt%. Furthermore, devices manufactured using PVK as host polymer instead of F8BT show 10 times lower EQE than OLEDs based on F8BT blends, also due to the lower PL quantum yield of 34 ± 3% (see Fig. S4 of the Supporting Information).

Beside the EL efficiency, a crucial aspect for practical application of NIR-OLEDs is their stability under continuous operation. In this regard, the performances of NIR-OLEDs based on phosphorescent dyes reported so far were affected by severe reduction of the EQE at high current densities (also known as ‘efficiency roll-off’). Efficiency roll-off originates from a number of quenching mechanisms including triplet-triplet annihilation, triplet-polaron quenching and electric-field-induced exciton dissociation facilitated by long emission lifetimes and intermolecular aggregation^43^. Remarkably, our **DPPcy**-based NIR-OLEDs exhibit essentially no efficiency roll-off with EQE ~ 0.5% for current densities as high as 50 mA/cm^2^, which is comparable to the best reported phosphorescent OLEDs based on Ir(III)-complex emitting in the same spectral region^24^,^44^.

Finally, to test the stability of **DPPcy** during continuous device operation, we biased our NIR-OLEDs at constant current density *J* = 10 mA/cm^2^ for 60 hours. Figure 4d shows that the EL intensity remains over 50% its initial value for the whole investigated time. Such a long device lifetime, which has never been reported for NIR-OLEDs based on pure organic emitters, derives directly from the inherent stability of **DPPcy** and further benefits from the low electrical stress due to well-balanced concentration of charge carriers in the thermally treated film. We finally highlight that all reported OLED performances were obtained with unprotected devices, and that these LEDs were not optimized in terms of layer thickness and electrodes work function, and that significant improvements might therefore still be expected by optimizing the electron-hole balance, e.g., by facilitating injection of the minority carriers^45^.

In conclusion, we have demonstrated the first example of NIR-OLEDs based on a NIR emitting diketopyrrolopyrrole borondifluoride cyanine emitter. This compound represents a valuable alternative to existing NIR chromophores for all-organic NIR-OLEDs emitting in the transparency window of biological tissues, which is particularly interesting for bioimaging and photodynamic therapy technologies. By incorporating our DPP derivative into a conductive polymeric matrix, we obtained a EQE = 0.55%, no efficiency roll-off and remarkable stability for as long as 60 hours of continuous operation. This new class of NIR electroluminescent materials, represented by, yet not limited to, the present diketopyrrolopyrrole derivative, will significantly contribute to the development of efficient all-organic NIR-OLEDs.

## Additional Information

**How to cite this article**: Sassi, M. *et al*. Near-infrared roll-off-free electroluminescence from highly stable diketopyrrolopyrrole light emitting diodes. *Sci. Rep.* **6**, 34096; doi: 10.1038/srep34096 (2016).

## Supplementary Material

Supplementary Information

## Figures and Tables

**Figure 1:**
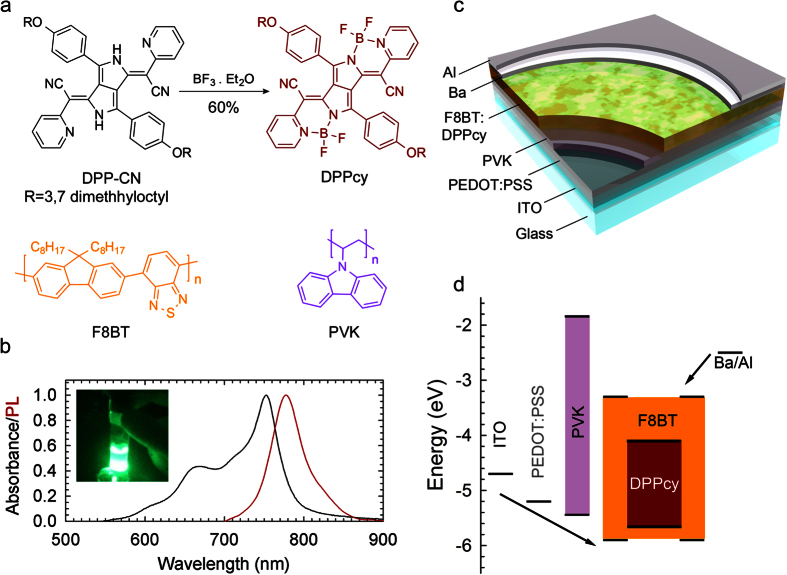
(**a**) Chemical structures of the DPP derivatives and the semiconductive polymers used for the fabrication of the NIR-OLEDs. (**b**) Absorption (black line) and photoluminescence (red line, excitation at 620 nm) spectra of a spin-coated film of **DPPcy** on glass. Inset shows a photograph of **DPPcy** in toluene taken with a UV-Vis filtered camera under UV illumination at 400 nm. (**c**) Device structure of a multilayer NIR-OLED: ITO/PEDOT:PSS/PVK/F8BT:**DPPcy**/Ba/Al. (**d**) Flat-band energy level diagram of the device (with respect to the vacuum level).

**Figure 2:**
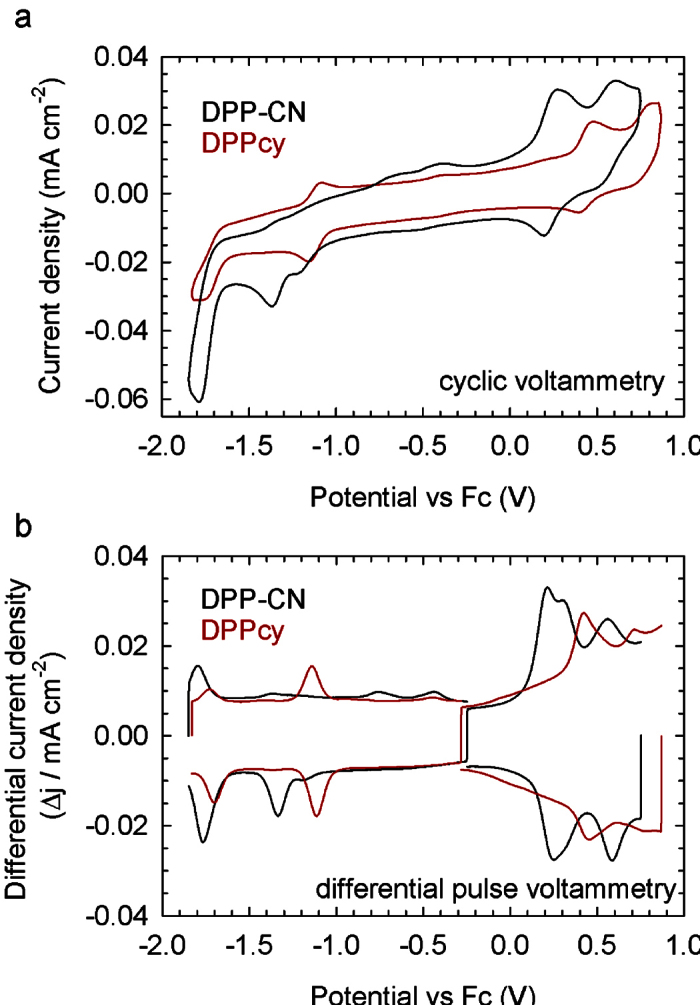
(**a**) Cyclic voltammetry and (**b**) differential pulse voltammetry traces of **DPP-CN** (black lines) and **DPPcy** (red lines) in a 2:1 CH_3_CN:CH_2_Cl_2_ solution with tetrabutylammoniumtetrafluoroborate 0.1 M as the supporting electrolyte.

**Figure 3:**
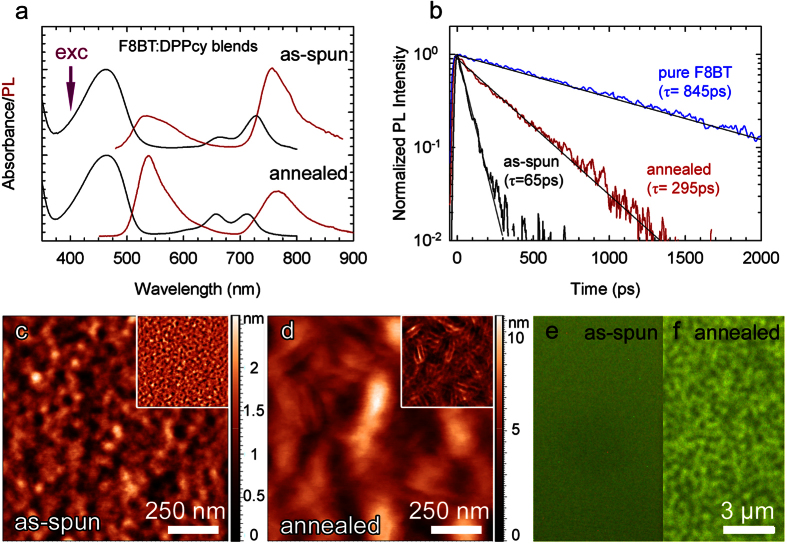
(**a**) Absorption and PL spectra under 405 nm excitation (indicated by a purple arrow) of F8BT:**DPPcy** (85:15 wt%) blended films as-spun (top curves) and thermally annealed at 130 °C for 15 minutes in nitrogen (bottom curves). (**b**) Time decay curves of F8BT photoluminescence at 535 nm for as-spun (black line) and thermally annealed (red line) films. The photoluminescence decay curve of a film of pure F8BT at 535 nm is reported as a blue line for direct comparison with the blended films (excitation at 405 nm). The single exponential fit of the decay curves are shown as solid black lines. Height- and phase- (inset) mode AFM images of (**c**) as-spun and (**d**) thermally annealed F8BT:**DPPcy** films. The white bar corresponds to 250 nm. (**e,f**) Respective fluorescence microscope images. The white bar corresponds to 3 μm.

**Figure 4:**
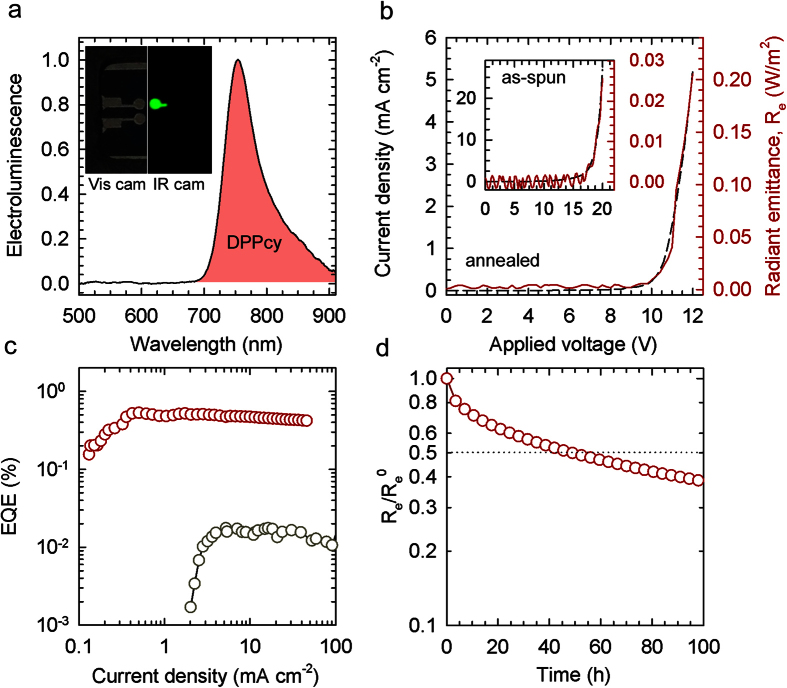
(**a**) EL spectrum of an ITO/PEDOT:PSS/PVK/F8BT:**DPPcy**(85:15 wt%)/Ba/Al OLED incorporating a thermally annealed active layer. A photograph of a working OLED collected with a UV-Vis filtered IR camera is reported in the inset, showing uniform bright IR emission over the whole device area (driving bias 5.5 V). (**b**) *J*-R_e_-V response of the same device and (inset) of an OLED with the same architecture embedding a pristine blend. (**c**) EQE vs. *J* of OLEDs incorporating pristine (grey circles) and annealed (red circles) films of F8BT:**DPPcy** 85:15 wt% blends. (**d**) Evolution of R_e_ with the device operation time at constant *J* = 10 mA/cm^2^ for an OLED embedding the annealed blend.
