# Supplementary material for: Near-infrared roll-off-free electroluminescence from highly stable diketopyrrolopyrrole light emitting diodes
Source: Sci Rep. 2016 Sep 28;6:34096. doi: 10.1038/srep34096 (PMC5039715; doi:10.1038/srep34096)

Supporting Information

Near-infrared roll-off-free electroluminescence from highly stable diketopyrrolopyrrole light emitting diodes

Mauro Sassi,[1] *Nunzio Buccheri*,[1] Myles Rooney,[1] *Chiara Botta,*[2] *Francesco Bruni,*[1] *Umberto Giovanella*,[2] *Sergio Brovelli*[1]* and *Luca Beverina*[1]*

[1] Dipartimento di Scienza dei Materiali Università degli Studi di Milano-Bicocca via Cozzi 55, 20125, Milano, Italy. Luca.beverina@unimib.it

[2]Istituto per lo Studio delle Macromolecole, Consiglio Nazionale delle Ricerche (ISMAC-CNR), Via Bassini 15, 20133, Milano, Italy.

1. Methods
2. Synthesis of DPP derivatives
3. Details on AFM and confocal optical measurements
4. TGA characterization of DPPcy
5. PVK based devices
6. OLEDS with different F8BT/DPPcy blending ratios
7. NMR spectra for derivative DPP-CN and DPPcy

1. Methods

Materials. All chemicals and solvents where purchased from Sigma Adrich, TCI Europe and Sigma. They where all used as received and not further purified. NMR spectra were recorded with a Bruker AMX 500 Avance operating at 500 MHz. Melting points were determined using an OPTIMELT MV-160 apparatus and are uncorrected. Chromatographic purifications were performed using Merck 9385 silica gel, pore size 60 Å, (230- 400 mesh). Derivative DPPcy was prepared according to the process shown in Scheme S1 of the Supporting Information.

Electrochemical Measurements. Both organic solvents and electrolyte salts were purchased anhydrous from Sigma Aldrich and stored under Argon atmosphere in a M-Braun glove box (O_2 sensor sensitivity <1 ppm). All organic based electrolyte experiments, both depositions and characterizations, were done in the glove box while water based experiments were performed in ambient atmosphere. Electrochemical cell was a three electrodes two compartments glass cylinder flask. Working, counter and reference electrodes were made of Au (0.0314 cm^2 active area) or Glassy Carbon (0.0707 cm^2 active area) pin, Platinum mesh and Ag/AgCl, respectively. A Princeton Applied EG&G PAR2273 potentiostat has been used for electrochemical measurements. Prior to a set of measurements in organic media, the Ag/AgCl pseudo reference electrode was calibrated using a Ferrocene 1 mM solution in the corresponding organic electrolyte. Fc^+/Fc couple is +0.69V vs. NHE.

Optical spectroscopy. The absorption spectra were recorded using a Perkin Elmer Lambda 900 spectrometer. The photoluminescence measurements were performed using a the second harmonic of a Ti:sapphire laser at 400 nm as excitation source and collecting the emitted light with a charged coupled device coupled to a spectrometer. The PL quantum efficiency measurements were performed coupling the same setup to an integrating sphere. Time resolved photoluminescence experiments were conducted using the same excitation source and collecting with a Hamamatsu streak camera with time resolution better than 7 ps. All PL measurements were performed with power density of 40 nJ/cm^2. All measurements were carried out at room temperature.

Devices preparation. A blend of F8BT:DPPcy with mass ratio (85:15) was dissolved in toluene 15 mg/mL. Indium tin oxide (ITO; 15 Ω cm^{-2}) substrates were cleaned by sonication in distilled water, acetone, and isopropyl alcohol. After treatment with nitrogen plasma, a thin film (40 nm) of filtered (nylon 0.45 μm) poly-(3,4-ethylenedioxythiophene)-poly(styrenesulfonicacid) (PEDOT:PSS, Clevios P VP AI 4083, H.C. Starck) used as a hole-injection layer at the anode interface was spin-coated onto the ITO substrate and then dried under nitrogen atmosphere at 150 °C for 15 minutes. A ~100 nm film of the emitting blend was then spin-coated. A thin layer of barium (4 nm) and subsequently a layer of Al (80 nm) were deposited on the top by vacuum ($5x10^{-7}$mbar) thermal evaporation. In the optimized architecture a 50 nm thick PVK layer is inserted between PEDOT:PSS and the active layer.

2. **Synthesis of the DPP derivatives**

Derivative **DPPcy** was prepared according to the process shown in Scheme S1. Specifically, condensation of two equivalents of the nitrile 1 with diisopropylsuccinate gave the diketopyrrolopyrrole precursor 2 according to the standard preparation protocol for this class of dyes. Two equivalents of 2-pyridylacetonitrile were than condensed with 2 in the presence of $POCl_3$, to give ligand 3 in 75 % yield. The later was then treated with excess $BF_3.Et_2O$ in refluxing chlorobenzene and in the presence of ethyldiisopropyl amine. Removal of all volatiles and a simple work up in water gave the final compound in 60 % yield.

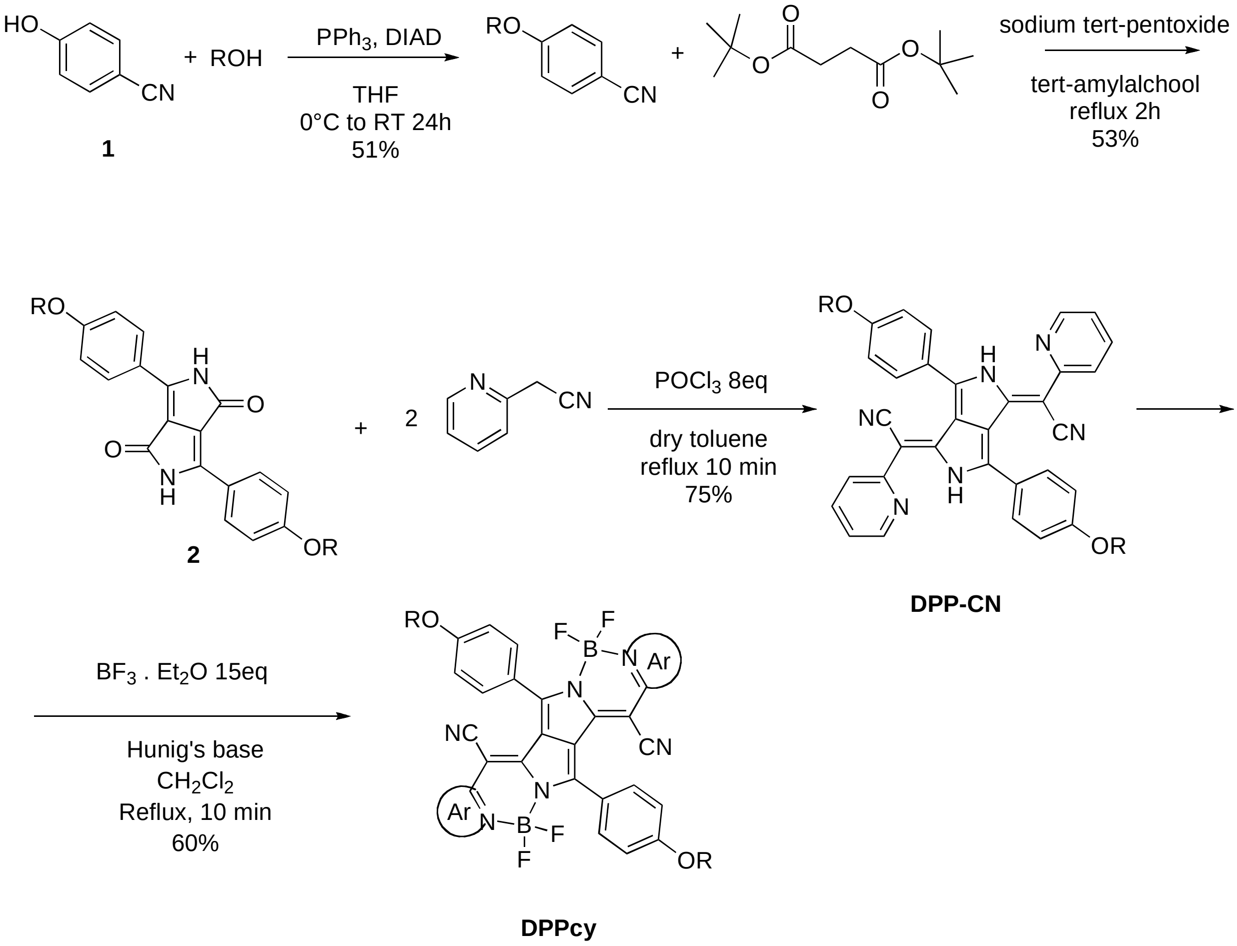

R= 3,7 dimethyloctyl

Scheme S1.

Synthesis of 4-(3,7-dimethyloctyloxy)benzonitrile.

4-cyanophenol (125 mmol) and triphenylphosphine (157.35 mmol) were dissolved in THF (200 ml) under nitrogen atmosphere at 0°C. A solution of 3-7-dimethyloctanol (104.9 mmol) in THF (200 ml) were then added and a mixture of DIAD (157.35 mmol) and THF 1:1 in volume was added dropwise over a hour. The reaction was stirred for 24 hours at R.T. The solvent was removed in vacuum and the residue brown oil was dissolved in CH_2Cl_2 (200 ml)and washed with a NaOH solution (5% w/w, 3x 150 ml). A solution of H_2O_2 (10% w/w, 60 ml) was added to the organic layer and the mixture stirred overnight. A solution of NaHSO3 (5% w/w, 100 ml) was then added and the organic phase was separated, the solvent removed, and dried over Na_2SO_4. The oil was triturated with hexane (300 ml) and the white precipitate formed was filtered off. A further purification was carried out with flash

chromatography (silica gel, eluent hexane, then 3:1 hexane Et_2O) affording the product as a brown oil (43.8 mmol, 51% yield).

Synthesis of 3,6-bis(4-(3,7-dimethyloctyloxy)phenyl)pyrrolo[3,4-c]pyrrole-1,4(2H,5H)-dione:

Sodium tert-pentoxide (65 mmol) and **1** (50mmol) were dissolved in tert-amylalcohol (150 ml) and heated at 125°C. A solution of di-tert-butylsuccinate (21,7 mmol) in the same solvent was added dropwise over 1.5 hours, then the reaction was refluxed for 2 hours. The mixture was cooled and isopropanol (20 ml) and water (50 ml) were added. The solid residue was filtered off and washed several times with hot methanol, then it was dried in vacuum at 50°C, giving the pure product as a red solid (11.5 mmol, 53% yield).

Syntesis of DPP-Cy N-H

$POCl_3$ (8eq) was added to a mixture of DPP (1eq) and pyridylacetonitrile (2.5 eq) in absolute toluene (20 ml/eq of DPP) at reflux in a nitrogen atmosphere. The reaction was monitored by TLC. After removal of the toluene and excess of $POCl_3$ by vacuum distillation, the crude product was dissolved in CH2Cl2 and neutralized with aqueous $NaHCO_3$ solution. The organic phase was separated and dried with $MgSO_4$. After removing the solvent, the residue was dissolved in acetone in an ultrasonic bath. The remaining solid was separated by filtration and washed several times with acetone giving a metal-like brown powder, 75% yield, ^{1}H NMR (500 MHz, $CDCl_3$): δ= 8.44 (d, J=4.5 Hz, 2H), 7.64 (m, 6H), 7.57 (d, J=8.3 Hz, 2H), 7.08 (d, J=8.7 Hz, 4H), 6.99 (t, J=5.2 Hz, 2H), 4.10 (m, 4H), 1.87 (m, 2H), 1.5-1.7 (m, 8H), 1.35 (m, 7H), 1.15 (m, 7H), 0.97 (d, J=3.4 Hz, 6H), 0.89 (d, J=6.7 Hz, 14H); ^{13}C NMR (125.75 MHz, $CDCl_3$): δ= 162.00, 156.66, 148.15, 146.15, 143.80, 137.68, 132.11, 123.16, 121.88, 121.50, 119.64, 117.25, 114.81, 78.77, 67.40, 40.13, 38.20, 37.04, 30.76, 28.87, 25.56, 23.60, 23.50, 20.58; Elemental analysis calc. (%) for $C_{52}H_{60}N_6O_2$ [M=808.07 g. mol-1]: C 77.97, H 7.55, N 10.49; found: C 78.20, H 7.70, N 10.54;

Syntesis of DPP-Cy boron complex

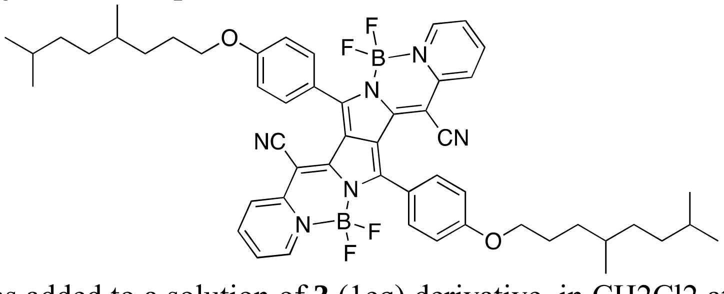

$BF_3.Et_20$ (15eq) was added to a solution of **3** (1eq) derivative in CH2Cl2 at reflux in nitrogen atmosphere. After 10 min, Hünig's base (2.5eq) was added and the mixture refluxed for further for further 10 min. The reaction was stopped and the solvent and $BF_3.Et_20$ excess was removed in vacuum. The crude solid was taken up with water and extracted with CH2Cl2. The organic layer was dried over Na2SO4, and evaporated after filtration, affording the boron complex as a green metallic solid in 60% yield, ^{1}H NMR (500 MHz, $CDCl_3$): δ= 8.32 (d, J=6.1 Hz, 2H), 7.86 (d dd, J_1=1.3, J_2=1.3, J_3=1.5 Hz 2H), 7.64 (d, J=8.6 Hz, 6H), 7.18 (t, J=6 Hz, 2H), 7.05 (d, J=8.7 Hz, 4H), 4.10 (m, 4H), 1.80-1.90 (m, 2H), 1.50-1.70 (m, 8H), 1.10-1.40 (m, 12H), 0.95 (d, J=6.5 Hz, 6H), 0.85 (d, J=6 Hz, 12H); ^{13}C NMR (125.75 MHz, $CDCl_3$): δ= 162.05, 154.36, 150.05, 146.81, 142.05, 141.58, 133.14, 123.18, 123.13, 122.62, 120.20, 116.87, 114.21, 67.18, 40.14, 38.22, 37.05, 30.75, 30.60, 28.90, 25.57, 23.64, 23.53, 20.62; ^{19}F NMR (376 MHz, $CDCl_3$): δ= 130.95; Elemental analysis calc. (%) for $C_{52}H_{58}B_2F_4N_6O_2$ [M=896.67 g. mol-1]: C 69.65, H 6.52, N 9.37; found: C 69.48, H 6.54, N 9.17;

3. Details on AFM and confocal optical measurements.

Atomic force microscopy (AFM) investigations were performed by using a NT-MDT NTEGRA instrument in semi-contact mode in ambient conditions.Microscopy Fluorescence images were collected with a Nikon Eclipse TE2000-U inverted confocal microscope. Excitation was obtained with a 100 W Hg lamp and 450-490 nm band-pass excitation filterusing a Plan Apo VC objective (magnification 100, N.A. 1.4).

4. TGA characterization of DPP-CN and DPPcy.

Traces acquired on a TGA/DSC 2 - ThermogravimetricAnalyzerMettler-Toledo at a heat scan ratio of 5°C/min under a nitrogen flux of 50 ml/min.

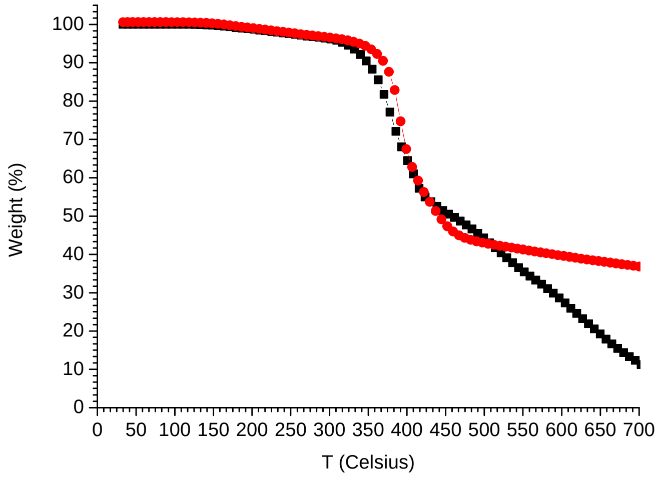

Figure S1. TGA traces under ambient (black squares) and nitrogen (red circles) atmosphere of derivative **DPPcy**. The behavior is very similar under both atmospheres with a well-defined weight loss starting at 340 °C.

5. DPP-based OLEDs in alternative device configurations.

We testedDPPcy as a NIR emitter in three different device configurations in additoin to the ITO/PEDOT:PSS/PVK/F8BT:**DPPcy**/Ba/Al structure reported in the main text : (1) OLEDs incorporating an active layer of pure DPPcy, (2) devices in ITO/PEDOT:PSS/PVK:**DPPcy**/Ba/Al architecture, in which the pure PVK hole transport layer and the F8BT matrix were replaced with a PVK:DPPcy blend and (3) dual layer OLEDs with structure ITO/PEDOT:PSS/PVK/PVK:**DPPcy**/Ba/Al. Apart from the metal contacts, all layers were deposed by spin coating similarly to the F8BT:**DPPcy** based OLEDs. OLEDs incorporating pure DPPcy as active layer showed no EL under any experimental conditions. Figure S1 shows representative EL spectra of OLEDs with structure(2) and (3).

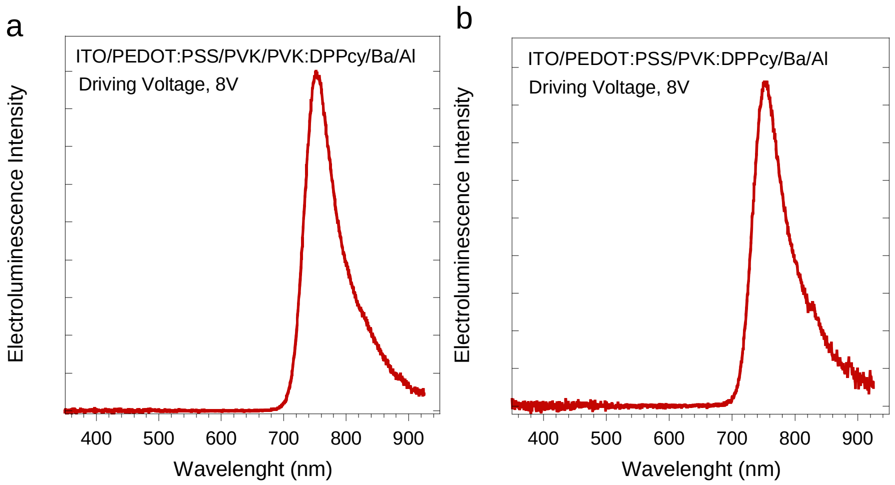

Figure S2. Electroluminescence spectra of a) dual layer ITO/PEDOT:PSS/PVK/PVK:**DPPcy**/Ba/Al OLED, b) single layer ITO/PEDOT:PSS/PVK:**DPPcy**/Ba/Al OLED.

In both cases we clearly observed ELpeaked at ~760 nm with FWHM of 65 nm, in very good agreement with the thin film PLspectrum. In both cases the efficiencies are, however, very low.

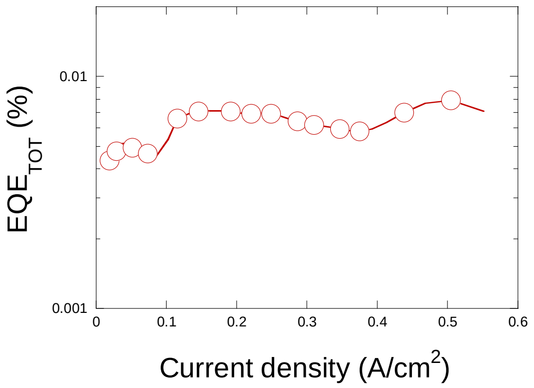

Figure S4. EQE for a ITO/PEDOT:PSS/PVK/PVK:**DPPcy**/Ba/Al device.

The EL is instead very stable. Figure S3 shows that after over 4 hours of continuous operation at 6 V, 80% of the original emission intensity is preserved. The inspection of the I-Vresponsessuggests large room for further optimization of the devices properties.

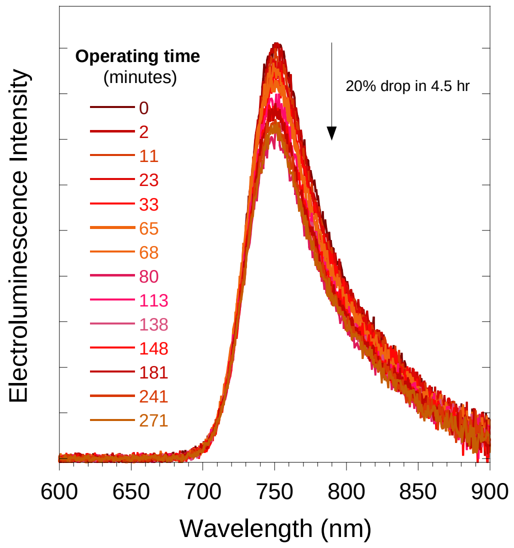

Figure S3. Electroluminescence spectra as a function of the continuous working time for a ITO/PEDOT:PSS/PVK/PVK:**DPPcy**/Ba/Al dual layer device.

The electroluminescence efficiency was in any case way lower that what observed when working with F8BT as the matrix. Figure S4.

6. Different F8BT/DPPcy ratios devices

Different F8BT/**DPPcy** ratios were tested, from 90:10 wt% to 70:30 wt%. The optimal doping level is 85:15 wt% that also corresponds to minimal contribution of F8BT in the EL spectrum.

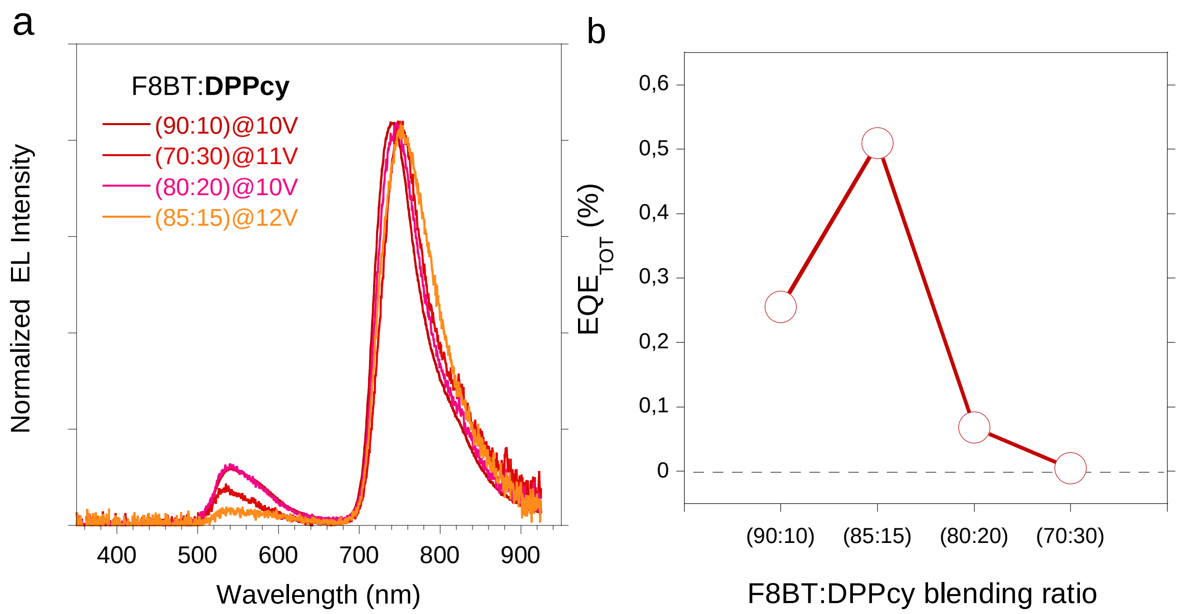

Figure S5. (a) Electroluminescence spectra of OLEDs based on a F8BT:**DPPcy** blends with different ratios. (b)Corresponding maximum EQE.

7. **NMR spectra for derivative DPP-CN and DPPcy**

a. **Derivative DPP-CN.**

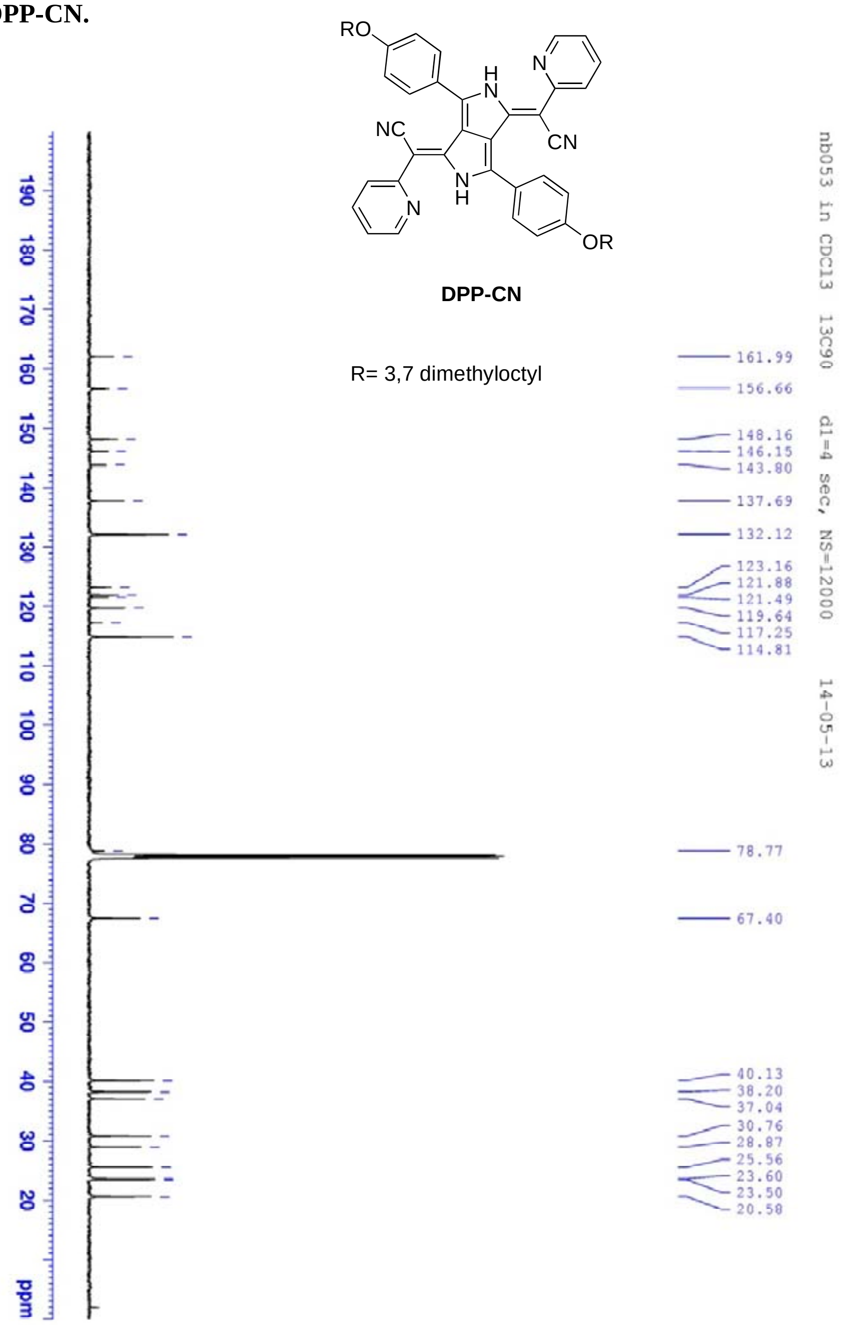

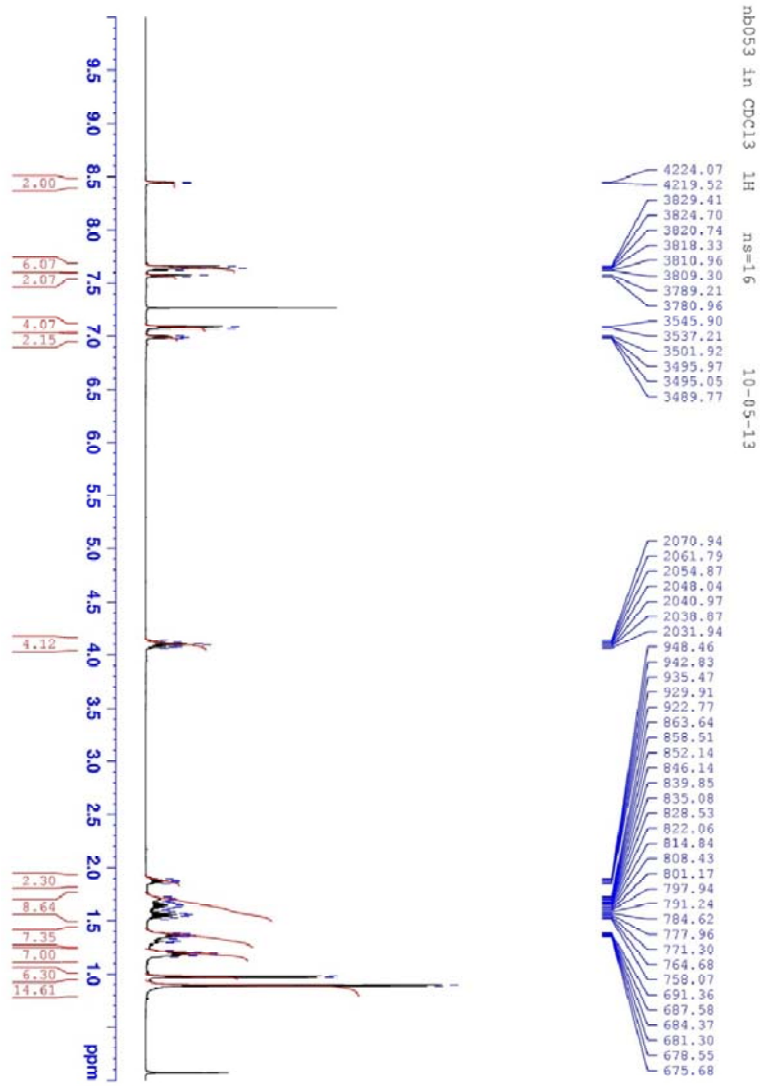
nb053 in CDCl3 1H ns=16 10-05-13
ppm
4224.07
4219.52
3829.41
3824.70
3820.74
3818.33
3810.96
3809.30
3789.21
3780.96
3545.90
3537.21
3501.92
3495.97
3495.05
3489.77
2070.94
2061.79
2054.87
2048.04
2040.97
2038.87
2031.94
948.46
942.83
935.47
929.91
922.77
863.64
858.51
852.14
846.14
839.85
835.08
828.53
822.06
814.84
808.43
801.17
797.94
791.24
784.62
777.96
771.30
764.68
758.07
691.36
687.58
684.37
681.30
678.55
675.68
2.00
6.07
2.07
4.07
2.15
4.12
2.30
8.64
7.35
7.00
6.30
14.61

a. Derivative DPPcy

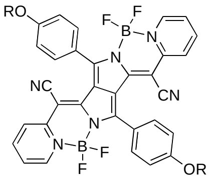

DPPcy

R= 3,7 dimethyloctyl

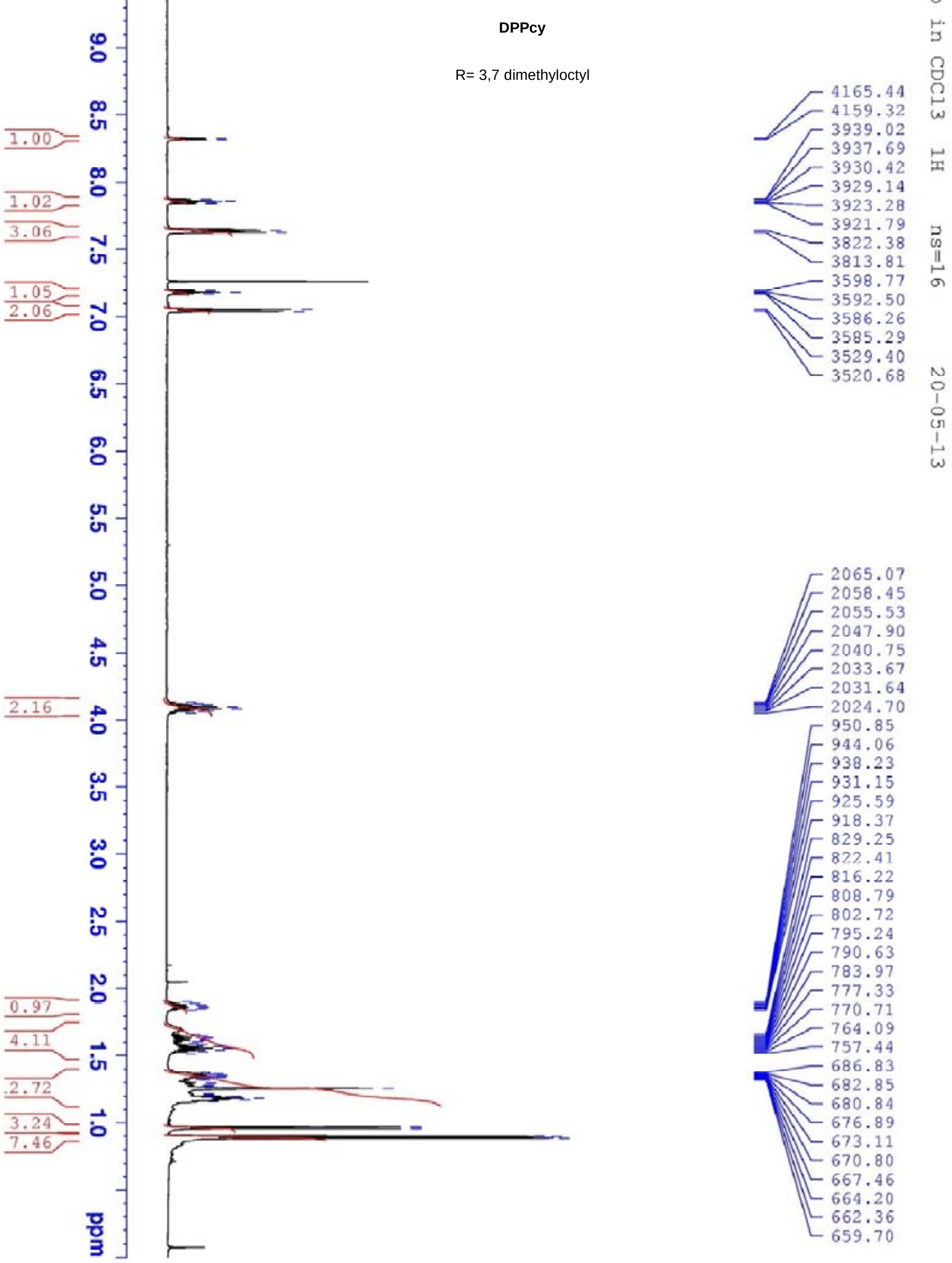

Supplement: Supplementary Information [file srep34096-s1.pdf]
